# Surgical outcomes of robotic thyroidectomy for thyroid tumors over 4 cm via the bilateral axillo-breast approach

**DOI:** 10.1038/s41598-024-62021-2

**Published:** 2024-05-21

**Authors:** Hye Lim Bae, Junice Shi-Hui Wong, Su-jin Kim, Younghoon Jung, Jae Bong Choi, JungHak Kwak, Hyeong Won Yu, Young Jun Chai, June Young Choi, Kyu Eun Lee

**Affiliations:** 1 Department of Surgery, Seoul National University Hospital and College of Medicine, Seoul, South Korea; 2 Department of Surgery, Tan Tock Seng Hospital, Singapore, Singapore; 3 Cancer Research Institute, Seoul National University College of Medicine, Seoul, South Korea; 4 Department of Surgery, Gachon University Gil Medical Center, Incheon, South Korea; 5 Division of Endocrine Surgery, Department of Surgery, Gibbeum Hospital, Seoul, South Korea; 6 Department of Surgery, Seoul National University Bundang Hospital, Seongnam, South Korea; 7 Department of Surgery, Seoul National University Boramae Medical Center, Seoul, South Korea; 8 Medical Big Data Research Center, Institute of Medical and Biological Engineering, Seoul National University, Seoul, South Korea

**Keywords:** Minimally invasive surgery, Robotic surgery, Bilateral axillo-breast approach, Thyroidectomy, Large goiter, Head and neck cancer, Thyroid cancer

## Abstract

The study investigated the feasibility of robotic bilateral axillo-breast approach (BABA) thyroidectomy for patients with thyroid tumors larger than 4 cm. BABA thyroidectomy has previously shown safety and effectiveness for thyroid surgeries but lacked extensive data on its application to larger tumors. Between October 2008 and August 2022, there were 74 patients underwent robotic BABA thyroidectomy due to thyroid nodules exceeding 4 cm in size. The mean patient age was 40.3 years. Fine needle aspiration results classified the tumors as benign (50.0%), atypia of undetermined significance (27.0%), follicular neoplasm (16.2%), suspicious for malignancy/malignancy (5.4%), or lymphoma (1.4%). The average tumor size was 4.9 cm, with the majority (85.1%) undergoing thyroid lobectomy, and the rest (14.9%) receiving total thyroidectomy. The mean total operation time was 178.4 min for lobectomy and 207.3 min for total thyroidectomy. Transient vocal cord palsy (VCP) was found in 3 patients (4.1%), and there was no permanent VCP. Among patients who underwent total thyroidectomy, transient hypoparathyroidism was observed in three (27.2%), and permanent hypoparathyroidism was observed in one (9.1%). There were no cases of open conversion, tumor spillage, bleeding, flap injury, or tumor recurrence. In conclusion, robotic BABA thyroidectomy may be a safe treatment option for large-sized thyroid tumors that carries no significant increase in complication rates.

## Introduction

Thyroid disease represents a prevalent disease worldwide, and open thyroidectomy is acknowledged as a standard procedure. Mu et al.’s recent review and meta-analysis revealed an incidence rate of 25% among individuals, indicating a notable upward trend in prevalence over the past decade^1^. In parallel, remote access surgeries for thyroidectomies have developed and are increasingly used in the last two decades. Since its initial introduction of endoscopic neck surgery by Gagner in 1996, various remote-access techniques worldwide have integrated endoscopic and robotic approach for thyroidectomy; transaxillary approaches (TAA), retroauricular approaches and transoral endoscopic thyroidectomy vestibular approaches (TOETVA)^2–6^. Their surgical applications have expanded to encompass the treatment of malignant tumors. Several studies have demonstrated the advantages of endoscopic and robotic thyroidectomy over conventional open thyroidectomy, including superior cosmetic outcomes, expedited return to normal activity, reduced incidence of postoperative complications, and enhanced surgical completeness^7–9^. This is particularly relevant as the majority of remote-access surgeries are performed for either benign conditions or small-sized differentiated thyroid cancer, both of which typically exhibit a favorable outcome.

Among remote access operations, the axillary-bilateral breast approach was first described in Japan in 2003^10^, followed by the development of the bilateral axillo-breast approach (BABA) in 2004 using endoscopic instruments^6^. The robotic platform was applied to this approach in 2008, and currently, robotic BABA thyroidectomy is one of the most popular remote-access techniques used in thyroid surgery^11^. In its early application, robotic BABA thyroidectomy was used for patients with small subcentimeter tumors. However, with increasing experience and expertise, the indications for this approach are expanding, including thyroid cancer (2–4 cm), differentiated thyroid cancer with lateral node metastasis, Graves’ disease, completion thyroidectomy and large thyroid tumors^12^. Nonetheless, there is a lack of data regarding the use of robotic BABA thyroidectomy for large thyroid nodules.

In this study, we aimed to analyze the clinicopathologic features and surgical outcomes of patients with nodules larger than 4 cm who underwent robotic BABA thyroidectomy. To our knowledge, this is the first study focusing on the feasibility and surgical outcomes of robotic BABA surgery for large nodules (> 4 cm).

## Results

### Patient demographics and clinicopathological characteristics

A total of 74 patients were included in the study, and the demographics and clinicopathological characteristics are shown in Table 1. The present study included 68 women (91.9%) and 6 men (8.1%), with a mean age of 40.3 ± 12.9 years. Thyroid nodules were diagnosed by fine needle aspiration (FNA) as benign (n = 37, 50.0%), atypia of undetermined significance (n = 20, 27.0%), follicular neoplasm (n = 12, 16.2%), malignancy (n = 4, 5.4%) and lymphoma (n = 1, 1.4%). The size of the dominant thyroid nodule was 4.9 ± 0.8 cm (range 4.0–8.5 cm) by preoperative ultrasonography. Thyroid lobectomy was performed in 63 patients (85.1%), while total thyroidectomy was performed in 11 (14.9%) patients. The cumulative postoperative drainage amounted to 149.3 ± 64.6 cc (range 24–312 cc), while the Visual Analog Scale (VAS) regarding postoperative pain averaged 3.0 ± 0.7 (range 1.6–5.3). The mean operation time was 182.7 ± 41.9 min, and the mean length of hospital stay was 2.9 ± 0.7 days.

**Table 1:** Patient demographic characteristics.

Patient demographic characteristics	Total (n = 74)
Sex (n, %)	
Female	68 (91.9%)
Male	6 (8.1%)
Age (years, mean $\pm$ SD)	40.3 $\pm$ 12.9 [15–67]
BMI (mean $\pm$ SD)	22.9 $\pm$ 3.7 [16.7–35.5)
Location of thyroid nodule (n, %)	
Right	35 (47.3%)
Left	39 (52.7%)
Preoperative nodule size (cm, mean $\pm$ SD)	4.9 $\pm$ 0.8 [4.0–8.5]
Preoperative FNA (n, %)	
Benign	37 (50.0%)
AUS	20 (27.0%)
FN	12 (16.2%)
PTC/suspicious PTC	4 (5.4%)
Lymphoma	1 (1.4%)
Extent of thyroidectomy (n, %)	
Lobectomy	63 (85.1%)
Total thyroidectomy	11 (14.9%)
Central lymph node dissection (n, %)	
No	65 (87.8%)
Yes	9 (12.2%)
Estimated blood loss (cc)	192.9 $\pm$ 166.9 [50.0–550.0]
Postoperative drainage amount (cc)	149.3 $\pm$ 64.6 [24–312]
Operation date	65.7 $\pm$ 31.6 [3–102]
POD#1	78.9 $\pm$ 22.3 [41.0–113.0]
POD#2	49.9 $\pm$ 20.3 [23.0–80.0]
POD#3	26.8 $\pm$ 17.7 [8–60]
Total amount	149.3 $\pm$ 64.6 [24–312]
Postoperative pain score (VAS)	
Operation day	4.3 $\pm$ 1.0 [3–6]
POD#1	3.1 $\pm$ 0.5 [2–4]
POD#2	2.4 $\pm$ 0.8 [1–4]
POD#3	2.4 $\pm$ 0.7 [1–3]
Length of hospital stay (days)	2.9 ± 0.7 [2–5]
Operation time (min)	182.7 ± 41.9 [90–310]
Thyroid volume (mm^3^)	42.8 ± 23.3 [11.1–132.1]
Thyroid weight (g)	34.7 ± 19.6 [10.5–136.0]

*BMI* body mass index, *FNA* fine needle aspiration, *AG* adenomatous goiter, *AUS* atypia of undetermined significance, *FN* follicular neoplasm, *PTC* papillary thyroid carcinoma, *EBL* estimated blood loss.

The final histopathologic results are shown in Table 2. The most common type of tumor was benign (n = 40, 54.1%), followed by high-risk neoplasms (n = 28, 37.8%) and low-risk neoplasms (n = 4, 5.4%). Among the patients with high-risk neoplasms, 13 patients underwent completion thyroidectomy according to American Thyroid Association 2015 guideline risk stratification^13^. All completion thyroidectomy cases were performed by robotic BABA thyroidectomy within 6 months from the first operation. The median follow-up period was 64.9 $\pm$ 29.2 months (range 4.0–170.0 months).

**Table 2:** Final histopathological results.

Histopathological results	Total (n = 74)
Benign tumor (n = 40, 54.1%)	
Follicular nodular disease	24 (32.4%)
Follicular adenoma	13 (16.2%)
Oncocytic adenoma	3 (4.1%)
Low-risk neoplasm (n = 4, 5.4%)	
FT-UMP	2 (2.7%)
NIFTP	1 (1.4%)
Hyalinizing trabecular tumor	1 (1.4%)
High-risk neoplasm (n = 28, 37.8%)	
Follicular thyroid carcinoma	14 (18.9%)
FVPTC	10 (13.5%)
PTC	2 (2.8%)
Oncocytic carcinoma	2 (2.8%)
ETC (n = 2, 2.8%)	
Dyshormonogenetic goiters	1 (1.4%)
Lymphoma	1 (1.4%)

*FT-UMP* follicular tumor of uncertain malignant potential, *FVPTC* follicular variant papillary thyroid carcinoma, *PTC* papillary thyroid carcinoma.

### Surgical outcomes

The operation time for each step is shown in Table 3. The mean total operation time was 178.4 ± 39.6 min for lobectomy and 207.3 ± 48.4 min for total thyroidectomy (Table 3, Supplementary Fig. 1). The mean total operation time and console time were significantly shorter in the robotic BABA lobectomy group than in the robotic BABA total thyroidectomy group (p = 0.033, p = 0.048). There were no significant differences between lobectomy and total thyroidectomy in each step mentioned as follows: robot setting and draping, flap dissection and closure time.

**Table 3:** Analysis of operation times between lobectomy and total thyroidectomy.

Operation step	Lobectomy	Total thyroidectomy	p value
Total operation	178.4 ± 39.6 (min)	207.3 ± 48.4 (min)	0.034
Robot setting and draping	23.3 ± 10.1 (min)	25.0 ± 8.5 (min)	0.613
Flap dissection	46.4 ± 15.0 (min)	45.0 ± 21.8 (min)	0.832
Console time	60.4 ± 27.6 (min)	94.0 ± 30.1 (min)	0.017
Closure	20.9 ± 7.8 (min)	20.0 ± 4.5 (min)	0.785

To assess the correlation between tumor size and operation time, we conducted a correlation analysis of the duration of each step involved in the surgical procedure (Table 4). As shown in Table 4, the tumor size had no effect on the total operation time or each operation step. In the case of lobectomy, no statistically significant correlations were found between the total procedure time and flap dissection time, with coefficients of 0.06 and 0.065 (*p* = 0.641, *p* = 0.673). As for total thyroidectomy, the study demonstrated the coefficients of 0.013 between the total operation time and tumor size, which was not statistically significant (*p* = 0.970).

**Table 4:** The correlation coefficient values between operation times and tumor size.

Operation step	Lobectomy	p value	Total thyroidectomy	p value
Total operation	0.060	0.641	0.013	0.970
Robot setting and draping	− 0.096	0.476	− 0.310	0.384
Flap dissection	0.065	0.673	− 0.206	0.657
Console time	− 0.003	0.986	− 0.122	0.845
Closure	− 0.061	0.714	− 0.278	0.594

There were no cases of open conversion, tumor spillage, bleeding, seroma, wound infection, flap injury or tracheal injury. Three patients (n = 3/74, 4.1%) showed transient vocal cord palsy, and all of them recovered within 6 months. In robotic BABA total thyroidectomy, there were 3 cases (n = 3/11, 27.2%) of transient hypoparathyroidism and 1 case (n = 1/11, 9.1%) of permanent hypoparathyroidism (Table 5). The final pathological analysis indicated malignancy in 28 patients, with 13 (n = 17/28, 60.7%) subsequently undergoing completion thyroidectomy and 17 (n = 17/28, 60.7%) receiving Radioactive Iodine treatment (Supplementary Table 1). There was no tumor recurrence during the follow-up period.

**Table 5:** Postoperative complications (n = 74).

Postoperative complication	n, %
Vocal cord palsy	
Transient	3/74 (4.1%)
Permanent	0/74 (0.0%)
Other complications	0/74 (0%)
Bleeding	0/74 (0%)
Seroma	0/74 (0%)
Wound Infection	0/74 (0%)
Flap injury	0/74 (0%)
Tracheal injury	0/74 (0%)
Hypoparathyroidism*	
Transient	3/11 (27.2%)
Permanent	1/11 (9.1%)

*Hypoparathyroidism was evaluated in 11 patients who underwent total thyroidectomy.

## Discussion

Since introduction of robotic BABA thyroidectomy in 2008, robotic BABA thyroidectomy has become one of the most remarkable remote-access approaches globally, particularly in Korea^11^. Over time, many surgeons have demonstrated the safety of robotic BABA thyroidectomy and have tried to overcome existing surgical criteria^8,14–16^. A recent study also suggested that surgical indications might extend up to 4 cm for malignant tumors and up to 8 cm for benign nodules^17^. Nonetheless, there is limited research on robotic BABA thyroidectomy for large tumors (Supplementary Table 2). The present study was designed to analyze the clinic-pathological characteristics and surgical outcomes of robotic BABA thyroidectomy in patient with tumors larger than 4 cm. We revealed that robotic BABA thyroidectomy showed acceptable operation times and complication rates compared to previous studies^16,18^.

There are various approaches that lead to a “scarless” neck in thyroid surgery: TAA, retroauricular approach, TOETVA, and BABA. Each approach presents distinct advantages and size criteria^17,19–22^ (Supplementary Table 3). Since its establishment by Ikeda in 2000, TAA has accumulated substantial research and progressively broadened its indication^23^. Notably, the recommended indication for TAA extends up to 6 cm for benign nodules and up to 4 cm for malignancies^24^. Although the retroauricular approach demonstrates feasible outcomes, the studies on this method remains limited and the definitive indications are yet to be established^8^. TOETVA enables shorter flap times and facilitates the removal of both lobes. However, transoral port and restricted flap range may impede the extraction of large thyroid glands^24^. Consequently, the indications are confined to 6 cm for benign nodules and 2 cm for malignant tumors.

Among the remote-access techniques, robotic BABA surgery offers several advantages in managing larger-sized thyroid tumors. The use of 3 working ports excluding the camera port makes it easier to achieve good retraction for larger glands. It also facilitates the identification of the recurrent laryngeal nerves and parathyroid glands on either side of the neck. In addition, robotic BABA operation utilizes 4 small incisions at the bilateral breast and axillary incisions, allowing a symmetrical “bottom-up” view of the neck which mimics that of conventional open thyroid surgery. A meta-analysis by Shan et al. indicated that robotic BABA thyroidectomy required longer operative times than open thyroidectomy, mainly due to additional time for flap creation and robot docking^25^. Conversely, with the sufficient flap dissection procedure, robotic BABA thyroidectomy is able to secure a wider working space, which allows for dissection of larger tumors with greater ease. The endo-wrist exhibits a wide range of motion, which facilitates easy retraction of the gland medially or laterally and reduces operator fatigue (Supplementary Fig. 2). Furthermore, the axillary skin is easily stretched to accommodate specimen extraction and, if needed, can be extended more with little compromise to cosmetic results^26,27^. The aforementioned features provide the rationale that BABA can be considered a safe and feasible option even for Graves’ disease^28^. As a result, the present study yielded no cases of tumor spillage during thyroidectomy procedures, and there were no recurrences among patients during a follow-up period averaging 64.9 months.

In one recent study, Chai et al.^16^ examined the stability of robotic BABA total thyroidectomy in 21 thyroid cancers larger than 2 cm by analyzing the operation time and complications compared with conventional open surgery. Additionally, they analyzed follow-up data such as thyroglobulin levels, and radioactive iodine uptake to demonstrate the oncological outcomes. The mean operative duration was 165.1 ± 43.9, and the mean hospitalization stay was 3.2 ± 0.6 days. Major surgical complications included vocal cord paralysis in 4 cases (4/21, 19.0%) and hypoparathyroidism in 4 cases (4/21, 19.0%), with no cases of open conversion. However, as the previous study focused on malignant nodules larger than 2 cm, the average nodule size was relatively smaller than that in this study (2.8 ± 0.3 cm vs. 4.9 ± 0.8 cm), which could be associated with a shorter operation time (165.1 ± 43.9 min vs. 182.7 ± 41.9 min).

Another study, conducted by Johri et al.^18^ in India, analyzed the patients who underwent BABA for large goiters categorized tumor size (< 6 cm vs ≥ 6.0 cm). While the present paper solely focused on robotic BABA thyroidectomy, this study included endoscopic BABA or unilateral axillo-breast approach procedures. Regarding lobectomy, the present study had longer operation times than the previous study, which could be attributed to the time required for robot docking (size < 6.0 cm, 152.0 ± 38.6 vs. 175.6 ± 39.4; size ≥ 6.0 cm, 184.3 ± 85.5 vs. 195.6 ± 38.2, Supplementary Table 4). However, both studies showed a similar operation time (206.4 ± 62.0 vs. 207.3 ± 48.4) in patients who underwent total thyroidectomy with tumor less than 6 cm. In terms of complications, the present study demonstrated low rates of complications except for one case of permanent hypoparathyroidism. Whereas four cases of open conversion were reported in the prior study, we did not encounter any cases of open conversion (4.0% vs. 0.0%). The results might indicate that the robotic approach could exhibit more stability than the endoscopic approach when conducting surgery for large-sized nodule, despite a potential increase in operation time.

In previous three studies, the mean operation time for robotic BABA lobectomy, and total thyroidectomy ranged from 160.9 to 236.3 min, and 189.6 to 310.1 min, respectively^29–31^. The mean operation time of lobectomy was 178.4 min, and the mean operation time of total thyroidectomy was 207.3 min. Considering that the mean size of the tumor ranged from 0.8 to 1.1 cm in the other studies, the operation times of the study were comparable. In the present study, total operation time, and console time were significant longer in robotic BABA total thyroidectomy compared to robotic BABA lobectomy. There were no significant differences were observed in the time taken for setting and draping, flap dissection, and closure steps. Furthermore, referring to the previous study^32^, we stratified surgeons into experienced and beginner group based on 50 cases, and analyzed the operation time in thyroid lobectomy. The experienced group demonstrated a significant reduction in console time, while no difference was observed in total operation time between two groups (Supplementary Table 5).

Despite the large tumor size, there were no longer hospital stays or increased complication rates compared to those of the previous studies, indicating that the mean hospital stay was 3.1–4.5 days for open, endoscopic, and robotic procedues^25,33^. In terms of drainage and pain, the results appear to be similar, compared to previous studies of BABA thyroidectomy even in cases of large goiters. While the cumulative drainage from prior papers is estimated to range 149–189.9 cc, the present study showed the comparable drainage volume of 149.3 ± 64.6 cc^34,35^. In the context of pain evaluation, previous research reported VAS ranging from 4.2 to 2.38. The present study found comparable or marginally elevated VAS in comparison^7,31^. The incidence of transient VCP (4.1%) in the present study was also comparable to reported rates of 0–6% for conventional thyroidectomy^36^ and represented an improvement compared to 20.3% in an early series of endoscopic BABA surgery^6^. The incidence of transient and permanent hypoparathyroidism for conventional thyroidectomy has been reported to be from 0.3 to 49% and 0 to 13%, respectively^36^. In previous research on endoscopic/robotic thyroidectomies, incidences of 0–30% and 0–4% have been reported for transient and permanent hypoparathyroidism, respectively^7,9,37^. The results of this study showed comparable complication rates of transient hypoparathyroidism (27.2%). Although the rate of permanent hypoparathyroidism was 9.1%, the result may be affected by limited sample size. Furthermore, no other complications were observed including postoperative hematoma, seroma formation, or skin flap-related complications. Additionally, no open conversions were required in our institution.

Despite the comparable results, the present study has several limitations. First, the restricted number of cases included in this study makes it challenging to assess the complications in comparison to other research. Second, as a retrospective study, the results led to selection biases and missing data. Third, the final pathology includes a wide variety of both benign and malignant conditions, with benign conditions being the majority. Hence, it is also difficult to analyze the adequacy of oncological clearance for larger malignant tumors in patients undergoing robotic BABA thyroidectomy. To our knowledge, however, this is the first study focused on evaluating the surgical safety and outcomes of robotic BABA thyroidectomy for larger tumors that includes an analysis of the time taken for each operative step and postoperative complications.

In conclusion, the study revealed that patients with larger thyroid tumors can also benefit from having a “scarless in the neck” surgery. Robotic BABA surgery is a safe technique that can be extended to such patients with no significant increase in complications, provided there is a sufficient level of surgical experience and expertise.

## Methods

### Patients

We retrospectively reviewed the electronic medical charts of patients who underwent robotic BABA thyroidectomy for thyroid nodules over 4 cm with preoperative neck ultrasonography in Seoul National University Hospital between January 2008 and August 2022. Robotic thyroidectomies were performed using the da Vinci robotic system (Intuitive Surgical, Sunnyvale, CA, USA, Supplementary Fig. 3). The extent of thyroidectomy was determined by fine needle aspiration (FNA) results, tumor size, tumor location, multifocality and underlying thyroid disease (Hashimoto’s thyroiditis or Graves’ disease).

**Figure 1:**
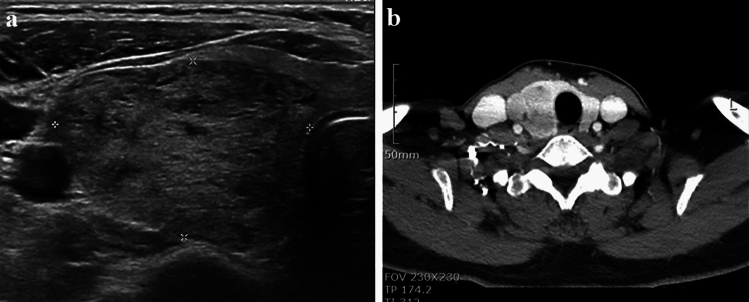
Preoperative ultrasonographic and neck computed tomographic image for an 56-year-old female patient with 4.2 cm goiter. (**a**) Ultrasonographic finding of thyroid goiter. There was suspicious 3.3 × 4.2 cm hypoechoic lesion in lower pole of right thyroid and no significant cervical lymph node enlargement. (**b**) CT finding of a 4.0 cm enhancing goiter in right thyroid gland.

All patients who underwent the operation were evaluated by preoperative sonography and CT (Fig. 1). The study was approved by the Institutional Review Board of Seoul National University Hospital (H-2212-056-1384). Informed consent was waived due to the retrospective nature of this study.

### Surgical procedure

**Figure 2:**
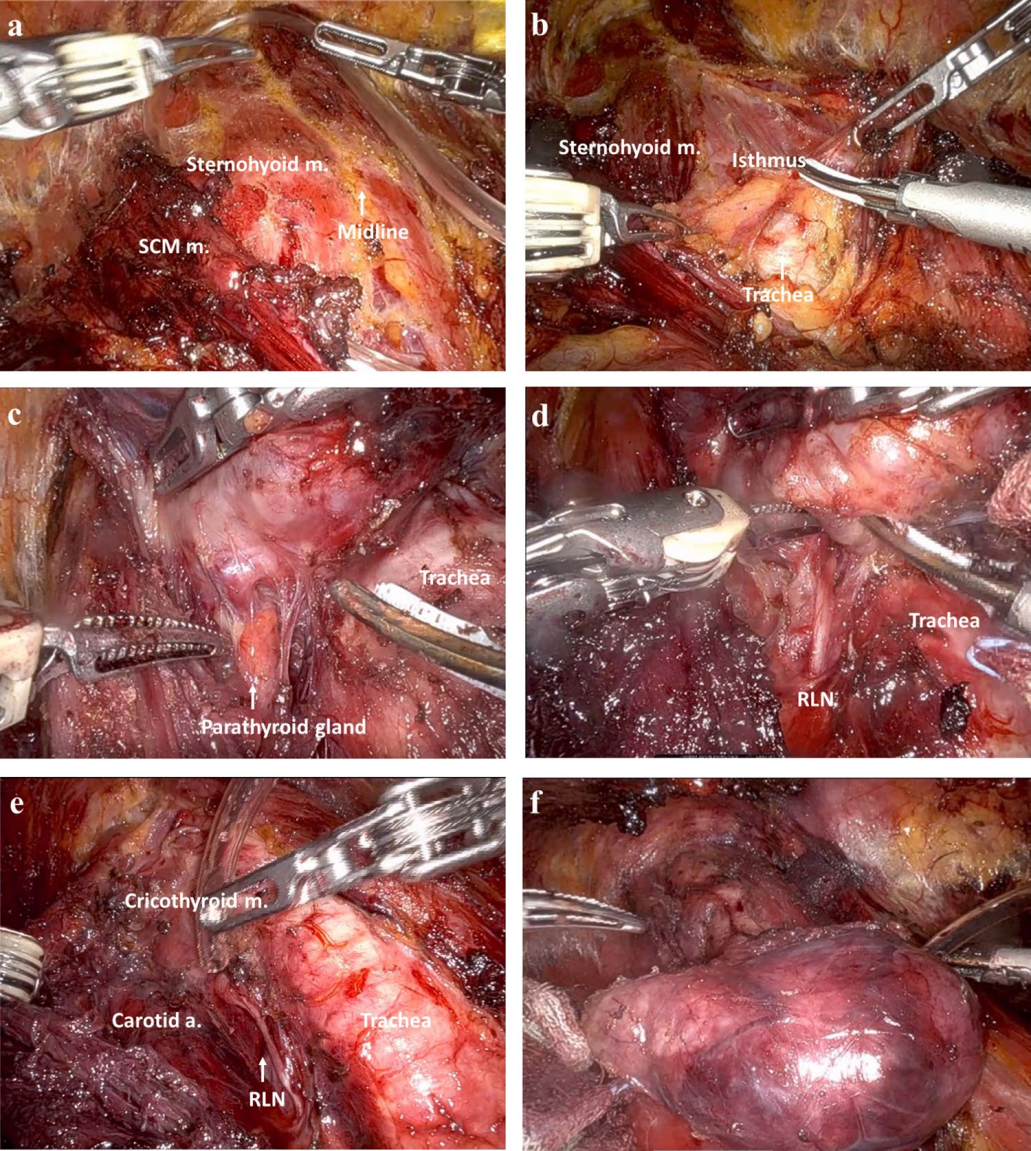
Robotic view of robotic BABA thyroidectomy. (**a**) Complete of the skin flap before thyroid lobectomy. (**b**) Midline division and isthmectomy at BABA robotic thyroid lobectomy. (**c**) Identification of parathyroid gland. (**d**) Identification of recurrent laryngeal nerve. (**e**) Completion of thyroid lobectomy. (**f**) Specimen. *SCM* sternocleidomastoid muscle, *m.* muscle, *RLN* recurrent laryngeal n.

The process of robotic BABA thyroidectomy involved the following steps: (i) setting and draping; (ii) flap dissection; (iii) thyroidectomy (console time); and (iv) closure^12^. (i) The setting and draping was defined as the induction of general anesthesia and the completion of surgical drape. Under general anesthesia, patients lay in the supine position with neck extension over the thyroid pillow, and surgeons draped from the neck to the breast area. (ii) Flap dissection was defined as the step from hydrodissection to completion of the skin flap. We drew the surgical marking over the chest for incision line and flap dissection. A diluted epinephrine solution (1:200,000) was injected along the surgical marking for hydrodissection. Four incisions were made for robotic BABA thyroidectomy in bilateral axillae and bilateral supra-areolas. In flap dissection, the operator made a working space from 2 cm below the clavicle to the thyroid cartilage and beyond the medial border of the sternocleidomastoid muscle. When creating the initial working space, blunt dissection was performed with a tunneler. After sufficient working space was made, the flap was extended by using an energy device. When flap dissection was complete, the position was modulated to 30 degrees of Trendelenburg, and robot docking started. (iii) In a routine robotic BABA thyroidectomy procedure, the midline was separated, and thyroidectomy was performed in console. In selected cases, lymph node dissection was conducted during the surgical procedure based on the results of FNA, imaging studies and the surgeon's clinical judgment. Throughout the surgical procedures, we attempted to locate and preserve the parathyroid glands. In all cases, an intraoperative neuro-monitoring system was used to identify and preserve recurrent laryngeal nerves (Fig. 2, Supplementary Fig. 4, Supplementary Video 1). (iv) The closure was executed by suturing the strap muscle and then closing the skin.

### Postoperative follow-up

The vocal cords of all subjects were evaluated using laryngoscopy or laryngeal ultrasonography preoperatively and postoperatively. VCP was diagnosed as fixation or hypomobility on laryngoscopy or sonography. For patients with vocal cord hypomobility or palsy, repeated examinations were conducted during the follow-up visits. Transient VCP was defined as an injury that recovered within 6 months, while permanent VCP was the status that did not recover beyond 6 months. In the case of total thyroidectomy patients, serum levels of total calcium, ionized calcium, phosphorus and parathyroid hormone were added to evaluate postoperative hypoparathyroidism. Hypoparathyroidism was defined by laboratory findings of parathyroid hormone under 15.0 pg/ml or the need for calcium medication for symptom relief, such as numbness and tingling sensations. Transient hypoparathyroidism was perceived as a recovery within 6 months, while permanent hypoparathyroidism was defined as still requiring calcium supplementation 6 months after the operation.

### Statistical analysis

Statistical analysis was performed using Statistic Package for Social Science (SPSS 25.0). Continuous variables are reported as the mean with standard deviation (SD), and categorical variables are reported as frequencies and percentages. While linear regression analysis was used to compare continuous variables, a paired T test was used to compare the categorical variables. Pearson’s correlation coefficients between tumor size and operation time were calculated.

### Ethics statement

The study was carried out in accordance with the principles laid out in the World Medical Association’s Declaration of Helsinki. This study was approved by the Institutional Review Board of Seoul National University Hospital. As data were obtained retrospectively, patient identities remained undisclosed. Informed consent is not mandatory for retrospective studies in Korea, and the institutional review board waived the need for informed consent.

### Supplementary Information

Supplementary Tables.

Supplementary Figures.

Supplementary Video 1.

Supplementary Legends.

## Data Availability

The datasets used and/or analyzed during the current study available from the corresponding author on reasonable request.

## Abbreviations

BABA: Bilateral axillo-breast approach
TAA: Transaxillary approaches
TOETVA: Transoral endoscopic thyroidectomy vestibular approaches
VAS: Visual analog scale
FNA: Fine needle aspiration
VCP: Vocal cord palsy
