# Supplementary material for: Surgical outcomes of robotic thyroidectomy for thyroid tumors over 4 cm via the bilateral axillo-breast approach
Source: Sci Rep. 2024 May 21;14:11646. doi: 10.1038/s41598-024-62021-2 (PMC11109269; doi:10.1038/s41598-024-62021-2)
Supplement: Supplementary file 1 — Supplementary Tables. [file 41598_2024_62021_MOESM1_ESM.docx]

**Supplementary Table 1. Oncological outcomes of patients with thyroid carcinoma.**

| ***Patient demographic characteristics*** | ***Total (n=28)*** |
| --- | --- |
| Permanent Pathology  Follicular variant PTC  PTC  MIFTC  WIFTC  Hurthle cell carcinoma  Minimally invasive oncocytic carcinoma | 10 (35.7%)  2 (7.1%)  11 (39.3%)  3 (10.7%)  1 (3.6%)  1 (3.6%) |
| Tumor size | 4.65 ± 0.9 [3.8-6.2] |
| Extrathyroidal extension | 2 (7.1%) |
| LN metastasis | 0 (0%) |
| T stage  II  III | 8 (28.6%)  20 (71.4%) |
| Completion thyroidectomy | 13 (46.4%) |
| RAI treatment | 17 (60.7%) |
| Thyroglobulin (ng/mL)  3 months  6 months  1 year  2 years | 3.74 ± 4.1 [0.1-14]  2.13 ± 3.0 [0.1-11]  1.77 ± 3.1 [0.1-13]  1.67 ± 3.1 [0.1-13] |

*PTC, papillary thyroid carcinoma; MIFTC, minimally invasive follicular thyroid carcinoma; WIFTC, widely invasive follicular carcinoma; LN, lymph node; RAI, radioactive iodine therapy*

**Supplementary Table 2. Clinical outcome of robotic surgery for large thyroid tumors**

| **Study, Year** | **Surgical approach** | **No. of patient** | **Size** | **Operation time**  **(mean ± SD), min** | | | **Complication** | | | | **Hospital stay**  **(mean ± SD), day** |
| --- | --- | --- | --- | --- | --- | --- | --- | --- | --- | --- | --- |
|  |  |  |  | **Lobectomy** | **Total thyoridectomy** | | **VCP** | **Hypo-**  **parathyroidism** | **Open**  **conversion** | |  |
| Chai YJ et al^16^,  2017 | Robotic BABA | 21 | 2 - 4cm | n/a | | 165.1 ± 43.9 | 4/21 (19.0 %) | 4/21 (19.0 %) | | 0 (0.0%) | 3.2 ± 0.6 |
| Johri G. et al^18^,  2018 | BABA, TOETVA | 61  40 | 2.5 - 6cm  6 - 11cm | 152.0 ± 38.6  184.3 ± 85.5 | | 206.4 ± 62.0  243 ± 57.92 | 5/61 (8.1%)  2/40 (5.0%) | 7/18 (38.9%)  11/20 (55.0%) | | 1/61 (1.6%)  3/40 (7.5%) | 3.4 ± 1.4  4.1 ± 1.2 |

*BABA, bilateral axillary breast approach; TOETVA, transoral endoscopic thyroidectomy vestibular approach; VCP, vocal cord palsy*

**Supplementary Table 3. Indications of robotic thyroidectomy (tumor size)**

| **Study** | **Year** | **Surgical approach** | **Size**  **(benign)** | **Size**  **(malignant)** |
| --- | --- | --- | --- | --- |
| JH Kwak et al^17^ | 2020 | BABA | $<$8cm | $<$4cm |
| Abramovici L et al^19^  Song CM et al^20^ | 2015  2013 | TAA | $<$5cm | $<$4cm |
| William SD et al^21^ | 2014 | Retroauricular approach | $<$4cm | n/a |
| Razavi CR et al^22^ | 2017 | TOETVA | $<$6cm | $<$ 2cm |

*BABA, bilateral axillary breast approach; TAA, transaxillary approach; TOETVA, transoral endoscopic thyroidectomy vestibular approach*

**Supplementary Table 4. Details of operative outcomes depending on the size of 6 cm**

| ***Parameter*** | **Size < 6.0cm (n=65)** | **Size ≥ 6.0cm (n=9)** |
| --- | --- | --- |
| Extent of thyroidectomy  Lobectomy  Total thyroidectomy  Completion thyroidectomy | 54 (83.1%)  11 (16.9%)  0 | 9 (100.0%)  0  0 |
| Operation time  Lobectomy | 175.6 ± 39.4 (min) | 195.6 ± 38.2 (min) |
| Total thyroidectomy | 207.3 ± 48.4 (min) | n/a |
| Completion thyroidectomy | n/a | n/a |
| Mean hospital stay (day) | 3.97 ± 0.73 | 3.56 ± 0.53 |
| Complication (n, %)  Vocal cord palsy  Transient  Permanent | 3 (4.6%)  0 (0%) | 0 (0%)  0 (0%) |
| Hypoparathyroidism  Transient  Permanent | 3 (27.2%)  1 (9.1%) | n/a  n/a |
| Other complications  Bleeding  Seroma  Wound Infection  Flap injury  Tracheal injury | 0 (0%)  0 (0%)  0 (0%)  0 (0%)  0 (0%)  0 (0%) | 0 (0%)  0 (0%)  0 (0%)  0 (0%)  0 (0%)  0 (0%) |

**Supplementary Table 5. Comparison of operation time between experienced and beginner groups in robotic BABA thyroid lobectomy.**

| ***Operation step*** | ***Experienced group***  ***(n=55)*** | ***Beginner group***  ***(n=8)*** | ***p value*** |
| --- | --- | --- | --- |
| Total operation | 180.0 ± 40.8 (min) | 168.4 ± 29.9 (min) | 0.223 |
| Robot setting & draping | 23.9 ± 10.5 (min) | 19.6 ± 6.4 (min) | 0.138 |
| Flap dissection | 56.7 ± 25.6 (min) | 40.8 ± 16.3 (min) | 0.168 |
| Console time | 56.7 ± 25.6 (min) | 96.7 ± 23.6 (min) | 0.007 |
| Closure | 21.3 ± 8.2 (min) | 18.0 ± 2.7 (min) | 0.189 |
