# Supplementary figures and images for: Surgical outcomes of robotic thyroidectomy for thyroid tumors over 4 cm via the bilateral axillo-breast approach
Source: Sci Rep. 2024 May 21;14:11646. doi: 10.1038/s41598-024-62021-2 (PMC11109269; doi:10.1038/s41598-024-62021-2)

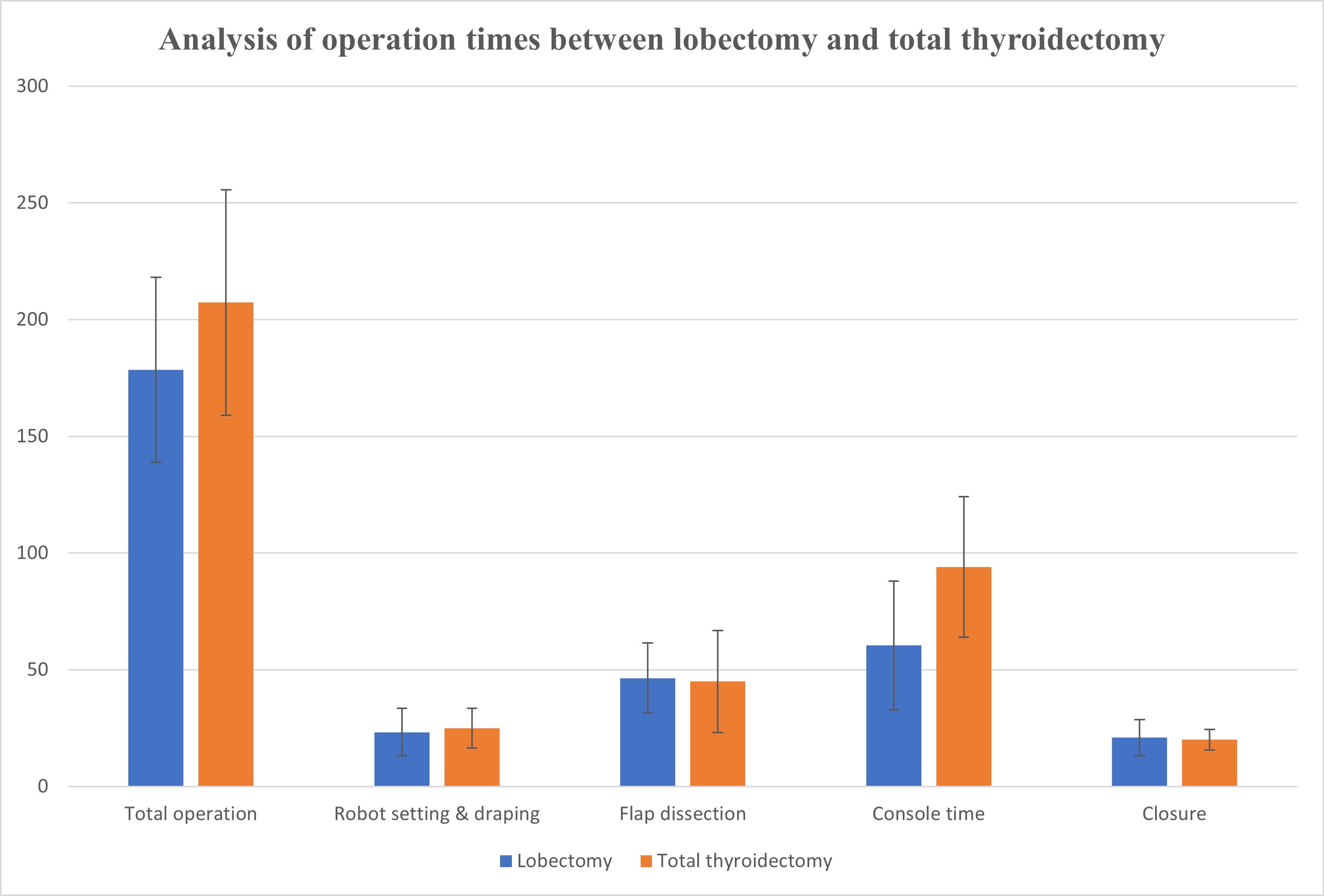

Supplement: Supplementary file 2 — Supplementary Figures. [file 41598_2024_62021_MOESM2_ESM.zip › Supple Fig 1_dpi300.jpg]

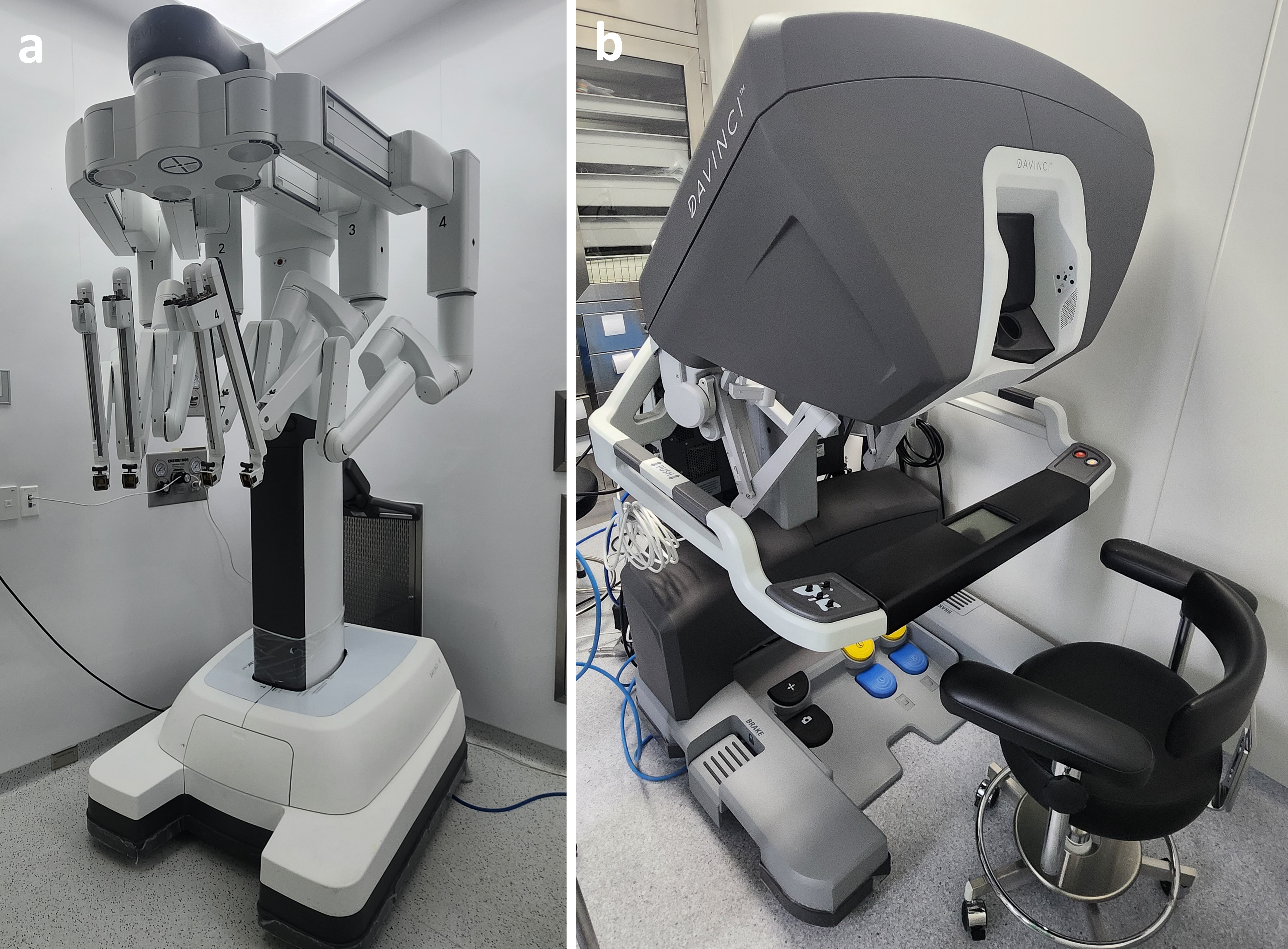

Supplement: Supplementary file 2 — Supplementary Figures. [file 41598_2024_62021_MOESM2_ESM.zip › Supple Fig 3ab_dpi300.tif]

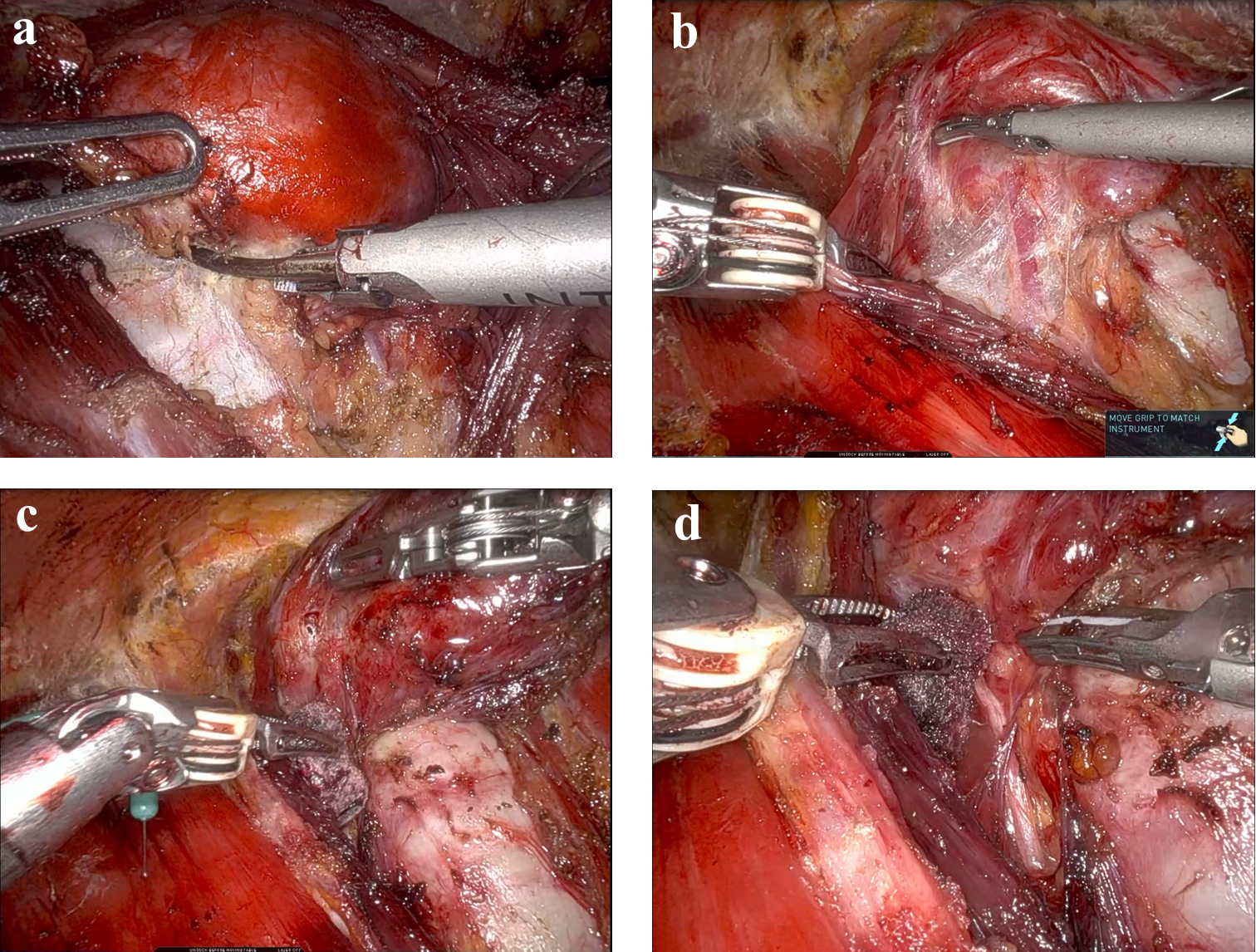

Supplement: Supplementary file 2 — Supplementary Figures. [file 41598_2024_62021_MOESM2_ESM.zip › Supple Figure 2_dpi300.tif]

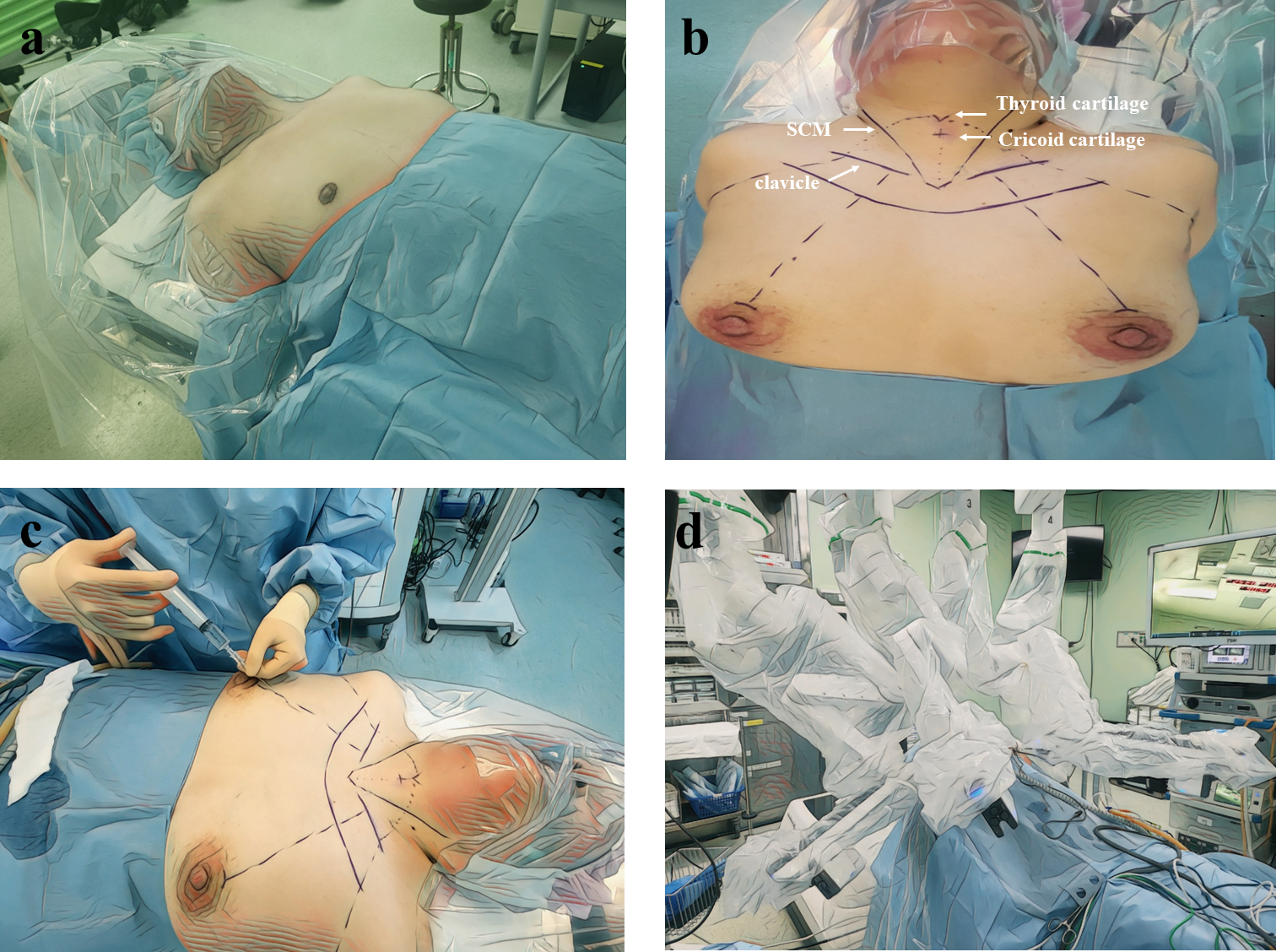

Supplement: Supplementary file 2 — Supplementary Figures. [file 41598_2024_62021_MOESM2_ESM.zip › Supple Figure 4 annot_dpi300.tif]
