# Supplementary material for: Surgical outcomes of robotic thyroidectomy for thyroid tumors over 4 cm via the bilateral axillo-breast approach
Source: Sci Rep. 2024 May 21;14:11646. doi: 10.1038/s41598-024-62021-2 (PMC11109269; doi:10.1038/s41598-024-62021-2)
Supplement: Supplementary file 4 — Supplementary Legends. [file 41598_2024_62021_MOESM4_ESM.docx]

**Supplementary Fig 1.** Analysis of operation times between lobectomy and total thyroidectomy. The mean total operation time and console time were significantly shorter in the robotic BABA lobectomy group than in the robotic BABA total thyroidectomy group (p=0.033, p=0.048).

**Supplementary Fig 2.** The figure is a surgical view of a 32-year-old female patient with a 4.3 cm -sized thyroid nodule, demonstrating the benefits of Robot BABA. (a) Dissection to separate the mass from the trachea (b) Elevation of the mass utilizing multirotational arms (c) Facilitation easy access the nodule through the broad flap (d) Identification of the recurrent laryngeal nerve, approaching by fine manipulation

**Supplementary Fig 3.** Da Vinci Xi (Intuitive Surgical, Sunnyvale, CA, USA) (a) patient cart (b) surgeon console

**Supplementary Fig 4.** Surgical procedure of robotic BABA thyroidectomy before docking (a) Positioning and draping (b) Sketching guideline along the landmarks. (c) Diluted epinephrine solution (1:200,000) is injected for hydrodissection (d) Robot docking

SCM, sternocleidomastoid muscle

**Supplementary Video 1.** Robotic BABA thyroidectomy for 4.2cm goiter
